# Blue Laser Light Counteracts HSV-1 in the SH-SY5Y Neuronal Cell Model of Infection

**DOI:** 10.3390/life12010055

**Published:** 2022-01-01

**Authors:** Luisa Zupin, Sergio Crovella

**Affiliations:** 1 Institute for Maternal and Child Health IRCCS Burlo Garofolo, 34137 Trieste, Italy; 2 Biological Science Program, Department of Biological and Environmental Sciences, College of Arts and Sciences, Qatar University, Doha 2713, Qatar; sgrovella@qu.edu.qa

**Keywords:** photobiomodulation therapy, HSV-1, blue wavelength

## Abstract

Herpes simplex virus 1 (HSV-1) is wide-spread virus that triggers painful and recurrent infections, as herpes labialis, causing blister lesions on the lip. HSV-1 infection can be a lifelong condition starting from childhood due to the latency of the virus hidden in the trigeminal ganglia. Despite the use of antiviral treatments, there is not a resolutive cure for herpes. In our study, we tested blue light against HSV-1 in a neuronal cellular model, aimed at mimicking the neuronal tropism of HSV-1. Two laser protocols employing continuous wave and pulse modalities were delivered to infected cell cultures and to the virus alone. A significant reduction of viral replication was observed when the beam was directly applied to the virus, along with an increase in cell survival. Our findings, considering the limitation of the still-unknown mechanisms by which the blue light acts on the virus, suggested a potential use of photobiomodulation therapy for clinical applications against herpes labialis in pediatric patients.

## 1. Introduction

Herpes simplex virus type 1 (HSV-1), a double-strand DNA virus belonging to the *Herpesviridae* virus family, is a wide-spread viral pathogen that is able to establish life-long infections in humans [1].

Generally, HSV-1 is mainly transmitted by mouth-to-mouth contact, through exposure to sores, saliva and surfaces in or around the mouth. Therefore, HSV-1 enters an organism through oral mucosa or damaged skin and replicates in the epithelial cells of the epidermis and dermis. From the first site of infection, HSV-1 can assault peripheral sensory nerve endings and establish a latent infection in neuronal cell bodies of the trigeminal ganglia; from here HSV-1 reactivation can result in the spread of the virus towards the muco-cutaneous region near the mouth. Gingivostomatitis and pharyngitis characterize the first infection, while virus reactivation provokes the recurrent herpes labialis, where cold sores, intraoral mucosal ulcerations/blisters on the mouth or on facial skin occur [1].

It has been estimated by the World Health Organization that immunoglobulin against HSV-1 are presented in about 90% of the population under 50 years old, however, recurrent infective episodes only arise in 67% of them [2]. Despite the incidence in the pediatric population decreasing, it has been assessed that 35% of children are HSV-1 seropositive. To should be noted that HSV-1 infection can be a life-long condition, that can start from childhood and persist until elderly age [3].

Anti-viral drugs, such as acyclovir, famciclovir and valacyclovir, are the first line of drug treatment, however, acyclovir-resistant strains have emerged and some patients are unable to adhere the treatment protocol (e.g., allergy); hence, the effect on recurrence of infection is reduced [4].

To should be noted that in immuno-compromised individuals, such as adults and pediatric oncologic patients undergoing radio-chemotherapy, the prevention of HSV-1 infection remains an issue to be solved [1,2]. Moreover, the current anti-viral treatment showed only a reduced effect in decreasing the occurrence of the infections [4].

Photobiomodulation therapy (PBMT) has already been reported as a promising form of treatment against HSV-1 infections, by using red, near-infrared or infrared wavelengths [5].

PBMT at red, near-infrared and infra-red wavelengths reduced the recurrence of infectious episodes in vivo on patients and contributed to the healing process of the HSV-1 oral vesicles [6]. Interestingly, Eduardo et al. treated patients with multiple PBMT sessions on oral and perioral areas in the prodromic stages when the virus was in a latent phase. The outcome at 3 years displayed a decrement in frequency of herpetic manifestations, with faster healing and milder symptoms [7]. Moreover, different works exploited an antimicrobial photodynamic approach, where an exogenous photosensitizer was used to elicit reactive oxygen species production, which led to virus inactivation [8]. Donnarumma et al. [9] observed that irradiated (λ 830 nm) HSV-1-infected HaCaT cells showed a reduction in viral replication and a decreased level of VP16, a tegument viral protein required for viral replication. Moreover, the authors observed an increment in pro-inflammatory cytokines and proteins, which are normally suppressed by progeny virions. They speculated that PBMT activated an immune, anti-viral response, blocking the final phase of HSV-1 replication. 

Among the wavelengths exploited in PBMT, the blue one is known for anti-microbial activity [10,11], also against viruses [12,13,14]. 

We previously reported the antiviral effect of blue laser light on HSV-1 in an epithelial cellular model of infection [13]; in the present study blue PBMT was tested against HSV-1 in vitro in a neuronal model of HSV-1 infection, with the aim of mimicking the neuronal tropism of HSV-1. 

## 2. Materials and Methods

### 2.1. HSV-1 Culture Infection

The SH-SY5Y neuroblastoma cell line (n. 94030304, Merck, Darmstadt, Germany), derived from a four-year-old, female children was selected as a neuronal model for HSV-1 infection, since the susceptibility of these cells to HSV-1 infection was previously observed [15,16]. 

The cells were maintained in 44.5% MEM and 44.5% Ham’s F12, supplemented with 10% fetal bovine serum, non-essential amino acid (10 mM for each amino acid), 2 mM glutamine and 100 U/mL penicillin/streptomycin (all the reagents were from Euroclone, Pero, Milan, Italy). A total of 10^4^ cells were seeded each day prior to infection in 96-multiwell plates (Sarstedt, Munich, Germany) in 100 μL of final volume. After 24 h they were infected with different quantities of viral DNA copies of HSV-1, i.e., 5 × 10^6^, 5 × 10^5^, 5 × 10^4^, 5 × 10^3^, and incubated for 24 h or for 48 h at 37 °C and 5% CO_2_. 

#### 2.1.1. Viability Assay

After 24 or 48 h the cells viability was determined with the 3-(4,5-dimethylthiazol-2-yl)-2, 5-diphenyltetrazoliumbromide (MTT) cell proliferation colorimetric assay (Trevigen, Gaithersburg, MD, USA), according to the manufacturer’s instructions. The absorbances were read in a GloMax^®^-Multi Detection System (Promega, Fitchburg, WI, USA).

#### 2.1.2. Viral DNA Quantification

At the same time points (24 and 48 h) the viral DNA in the supernatants were also assessed. HSV-1 nucleic acids were extracted with a ZR Viral DNA/RNA Kit™ (Zymo Research, Murphy Ave., Irvine, CA 92614, USA), according to the manufacturer’s instructions, and the DNA was quantified with primers and a probe against glycoprotein B gene (500 nM forward primer GCATCGTCGAGGAGGTGGAC, 500 nM reverse primer TTGAAGCGGTCGGCGGCGTA, 200 nM probe FAM CGACCCCTCCCGGTAGCCGT TAMRA (Merck) [17]), with the Kapa Probe Fast Universal One-step qRT-PCR kit (Kapa Biosystems, Inc. Wilmington, MA, USA) on a LightCycler^®^ 480 Real Time PCR Instrument II (Roche Molecular Diagnostics, F. Hoffmann-La Roche AG, Basel, Switzerland). The protocol of amplification was as follows: 95 °C for 3 min, 45 cycles with 95 °C for 3 s and 60 °C for 30 s, and finally 40 °C for 30 s. 

A homemade standard was employed. Briefly, after virus amplification on the Vero E6 cells (ATCC CRL-1586), the viral DNA was extracted from the supernatants with the ZR Viral DNA/RNA Kit™ (Zymo Research) and quantified with the diagnostic kit HSV1 Q—PCR Alert Kit (ELItechgroup, Puteaux, France). Serial dilutions of the extracted DNA were then used to generate the standard curve.

### 2.2. Laser Irradiation

The irradiation was carried out with a gallium arsenide (GaAs) plus indium gallium aluminium arsenide phosphide (InGaAlAsP) diode laser device (class IV, K-Laser Cube series, K-laser d.o.o., Sežana, Slovenia) with the following protocols: λ, 445 nm; irradiance, 0.1, 0.2, 0.3 W/cm^2^; and fluences, 3, 10, 20 J/cm^2^, in a continuous wave (CW) or pulsed modality (at 5 Hz, with 50% duty cycle) with a spot size of 1 cm^2^.

The irradiation was performed on 96-multiwell plates in a final volume of 100 μL of medium (DMEM without phenol red, BE12-917F, Lonza, Basel, Switzerland) for each well, without the cover during laser delivery. The laser emission zoom probe was kept perpendicularly above the bottom of the well and the irradiation was carried out in a hood in the dark to avoid light interference. The emitted beam entirely covered the wells of the culture plate, and the power delivered was checked using an optical power meter (LaserPoint Plus+, Milan, Italy) and adapted to achieve the desired irradiance. The control group was not exposed to a laser.

The PBMT was tested initially on SH-SY5Y cells to determine the maximal doses tolerated by the cells: after 24 h from irradiation the vitality was assessed with the MTT Cell Proliferation Assay (Trevigen, Gaithersburg, MD, USA) according with to the manufacturer’s instructions, and the absorbances were read in a GloMax^®^-Multi Detection System (Promega, Fitchburg, WI, USA). 

### 2.3. Antiviral Effect of PBMT

The PBMT antiviral properties were evaluated in two different experimental settings:

- Irradiation of HSV-1 (5 × 10^4^ viral copies)—the virus was treated and after 30 min transferred to the cells and maintained in incubator for 24 h.
- Irradiation of HSV-1 infected culture (5 × 10^4^ viral copies)—the cells were infected for 1 h and then irradiated.

After 24 h from the treatments, the cells vitality and the HSV-1 viral DNA concentration in the supernatants were assessed as described above.

The experiments were replicated 3 times.

### 2.4. Statistical Analysis

The Kruskal–Wallis test with Dunn’s multiple comparison test was performed on the R software [18] to calculate the differences among the experimental conditions. All statistical analyses were two-sided, and *p*-values < 0.05 were considered significant. 

## 3. Results

### 3.1. HSV-1 Cell Culture Infection 

To choose the concentration of HSV-1, four different dilutions of HSV-1 were tested. All the dilutions resulted in a productive infection with increments in viral DNA in the supernatants at 24 and 48 h, along with a decrement in cell survival. The dilution 5 × 10^4^ viral copies were selected based on the cells’ mortality of around 40%, with respect to the untreated cells (Figure S1A,B).

### 3.2. PBMT Protocols

The following protocols were tested to determine the doses tolerated by the cells: λ, 445; irradiance, 0.3, 0.2, 0.1 W/cm^2^; fluency, 10, 20 J/cm^2^ for each irradiance, both in continuous (CW) and pulsed (frequency = 5 Hertz, Hz) waves. As the protocols were cytotoxic for these types of cells, the fluence was decremented at 3 J/cm^2^. Therefore, two protocols were chosen: PBMT 1 0.1 W/cm^2^, fluency 3 J/cm^2^, 5 Hz; and PBMT 2 0.1 W/cm^2^, fluency 3 J/cm^2^, CW (Figure S2).

### 3.3. Irradiation of HSV-1

**Figure 1:**
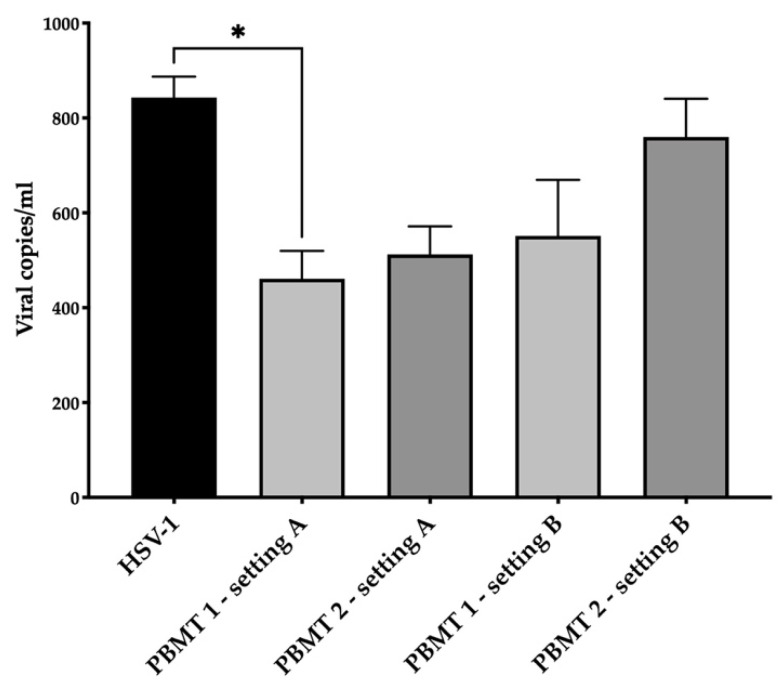
Antiviral effect of blue laser light on HSV-1 in the SH-SY5Y cell line. The viral DNA quantity in the supernatant after 24 h from treatment/infection was displayed. Two PBMT protocols were employed and designed as PBMT 1 (0.1 W/cm^2^, fluency 3 J/cm^2^, 5 Hz) and PBMT 2 (0.1 W/cm^2^, fluency 3 J/cm^2^, CW), in two experimental settings. In the first setting, A, which consisted of the irradiation of HSV-1, the virus was treated and after 30 min transferred to the cells and maintained in an incubator for 24 h. In the second setting, B, which consisted of the irradiation of the HSV-1-infected culture, the cells were infected for 1 h and then irradiated. The average (±standard deviation) viral load for mL of 3 replicates was reported. Results from the Kruskal–Wallis test adjusted by Dunn’s multiple comparison test were displayed (* *p* < 0.05).

**Figure 2:**
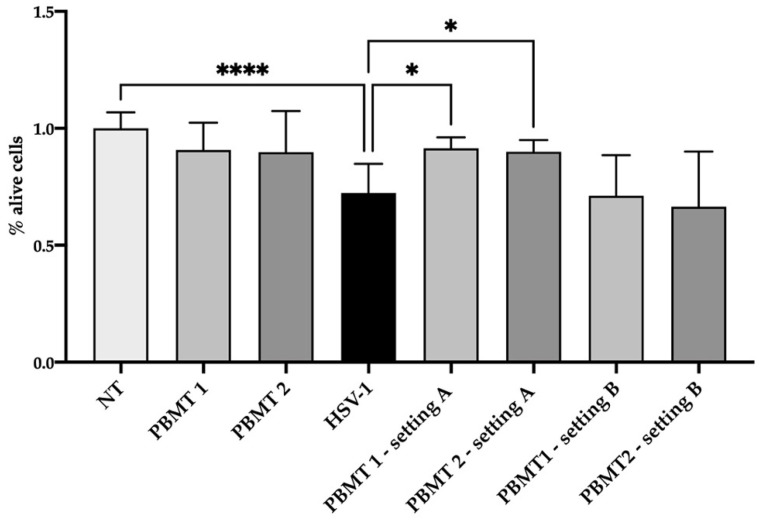
Viability of the SH-SY5Y cell line after 24 h from treatment/infection. The viability was displayed as a percentage of alive cells respect to not treated cells (NT). Two PBMT protocols were employed and designed as PBMT 1 (0.1 W/cm^2^, fluency 3 J/cm^2^, 5 Hz) and PBMT 2 (0.1 W/cm^2^, fluency 3 J/cm^2^, CW), in two experimental settings. In the first setting, A, which consisted of the irradiation of HSV-1, the virus was treated and after 30 min transferred to the cells and maintained in an incubator for 24 h. In the second setting, B, which consisted of the irradiation of the HSV-1-infected culture, the cells were infected for 1 h and then irradiated Three replicates of the experiments were performed. Results from Kruskal–Wallis test adjusted by Dunn’s multiple comparison test were displayed (* *p* < 0.05, **** *p* < 0.0001).

HSV-1 was diluted at a concentration of 5 × 10^4^ viral copies in 100 µL of culture medium (without phenol red) and irradiated with PBMT 1 and PBMT 2. After 24 h from treatment, the viral DNA quantity decreased (Figure 1) in the irradiated virus with respect to the untreated one in both protocols, although the statistical significance was only achieved with PBMT 1 (KW test, *p* = 0.01). Corroborating the result, the vitality of the cells infected with the virus and irradiated with both PBMT 1 and PBMT 2 protocols were comparable to the un-infected cells (KW test, *p* = 0.04 and *p* = 0.02, Figure 2).

### 3.4. HSV-1 Cell Culture Infection 

Cells were infected with HSV-1 (5 × 10^4^ viral copies in 100 µL) and then irradiated with PBMT 1 and PBMT 2. After 24 h, a trend of viral DNA decrement was observed (Figure 1), but the viability was at the same level as the cells infected with the non-irradiated virus (Figure 2).

## 4. Discussion

Our data showed that blue light had an antiviral effect on HSV-1, even at very low doses (irradiance 0.1 W/cm^2^, fluency 3 J/cm^2^).

The results highlighted direct PBMT activity on the virus itself with PBMT protocols employing continuous and pulsed waves, although the first (CW) was more efficacious in viral DNA abatement. The irradiation of the infected culture was less promising, with only a slight decrement in the viral DNA level, possibly suggesting that targeting the virus prior to cell entry was crucial in order to block the viral replication, while the PBMT effect when the virus was protected by the host cellular membrane was minor.

The use of blue light to counteract viruses is quite a recent application with respect to the wide-use of red and near-infrared light for biomodulation purposes [5], such as immunomodulation [19], analgesia [20,21] and wound healing [22].

Blue light at 445 nm was reported to be effective in vitro against different types of viruses, such as HSV-1 in an epithelial model (HaCaT cells) of cellular infection [13], where a decrement in the viral DNA and an increment in the cells’ survival was measured in the cells receiving the irradiated virus with protocol 0.15 W/cm^2^, 30 or 60 J/cm^2^, 5 Hz, which was much higher with respect to those employed in the current study (0.1 W/cm^2^, fluency 3 J/cm^2^). A similar, although less evident, result was observed when cells, already infected for 1 h, were irradiated (0.15 W/cm^2^, 30 or 60 J/cm^2^, 5 Hz). Blue PBMT at 445 nm (0.3 W/cm^2^, 60 J/cm^2^, CW) was also effective against the Zika virus (ZIKV), a positive-strand, RNA-enveloped virus, in a cellular glial model of infection (U87-MG cells) where, after blue PBMT, a decrement in the viral RNA was observed both when the virus was irradiated itself and then transferred to the cell culture and when cells were irradiated 1 h after infection. In the second scenario an increment in the cell viability was also observed [14]. In addition, in this case, the irradiance and fluence (0.3 W/cm^2^, 60 J/cm^2^) selected were higher with respect to those employed in the current work (0.1 W/cm^2^, fluency 3 J/cm^2^).

Enwemeka et al. [23] tested pulsed blue light at 405 nm, 410 nm, 425 nm and 450 nm (irradiance 3.0 mW/cm^2^ to 12 mW/cm^2^; fluence from 32.4 J/cm^2^ to 130 J/cm^2^) on human alpha coronavirus HCoV-229 E and human beta coronavirus HCoV-OC43. They determined a reduction in viral RNA concentration measured with a fluorometer and an RNA viral level quantified with Reverse Transcriptase Loop-Mediated Isothermal Amplification. However, they did not perform virus infection in the cell culture, for the assessment of residual infectivity.

Very recently, blue light was delivered to SARS-CoV-2 with very promising results. LED lamps at 450, 454 and 470 nm (CW) were able to cause a decrement in the viral RNA quantity when infected Vero E6 cells were irradiated (450 nm, 0.04 W/cm^2^; 12.5 J/cm^2^; 454 nm, 0.04 W/ cm^2^; 10 J/cm^2^; 470 nm, 0.04 W/ cm^2^, 20 J/cm^2^) and relevant decreases were reported, especially at 48 h post-treatment [12]. Stakos et al. [24] employed LED light with a peak at 425 nm (dose ranging from 7.5 to 60 J/cm^2^, 50 mW/cm^2^), on three coronaviruses, i.e., SARS-CoV-2, SARS-CoV-1 and MERS-CoV [24]. When both Vero cells and 3D human tracheal/bronchial-derived epithelial tissue were infected with SARS-CoV-2 and then irradiated, a reduction in viral infection was observed. Moreover, blue LED inhibited SARS-CoV-2, SARS-CoV-1 and MERS-CoV when the virus was irradiated alone and then transferred to Vero E6, Vero E6 and Vero CCL-81, respectively, but it was no effective on HRB-1 on Hela cells. This suggested that blue light possesses some degree of selectivity. Moreover, Oh et al. [25], showing the antiviral effect of blue light on SARS-CoV-2 already-infected cells (450 nm, 5.56 mW/cm^2^, 1.6, 5, 10 J/cm^2^), reported that an antiviral effect was associated with reactive oxygen species (ROS) production, endoplasmic reticulum stress and autophagy progression, inhibiting apoptosis and promoting cell survival. These studies employed minor irradiance with respect to those employed in the current study (0.1 W/cm^2^), nevertheless the fluence (3 J/cm^2^) was comparable [25] or higher [12,24]. Our results failed to highlight an antiviral effect in already-infected cells as the three papers cited above showed, however the different virus strains treated, and the different devices employed, could account for the results. Indeed, Oh et al. [25] observed a partial selectivity in the blue light’s action.

Two other works employed blue wavelength on SARS-CoV-2. De Santis et al. [26] obtained a viral inactivation delivering visible light to SARS-CoV-2, however, they used a very prolonged time of irradiation, i.e., 60 min, and a range of wavelengths from 400 to 780 nm, so a direct comparison with the other studies was not possible. 

Rathnasinghe et al. [27] explored the effect of 405 nm light with irradiance from 0.035 to 0.6 mW/cm^2^ on SARS-CoV-2, obtaining viral inactivation after at least 4 h of illumination. Although the irradiance was very low from that tested in the current study, and in the other research described above, the total energy reached very high levels.

Despite the great effort in the study of the direct antiviral properties of blue PBMT, its mechanism of action it still undefined.

Since the envelopes of enveloped viruses are generated from host cells’ membranes, it can be speculated that they contain endogenous photosensitizers (e.g., porphyrins, flavins, NADH), that can render the virus susceptible to blue light. A direct disrupting effect of light on the virus envelopes or the generation of virucidal ROS may be hypothesized [28].

## 5. Conclusions

Our results show that blue light was able, in vitro, to decrease the HSV-1 DNA quantity and improve the vitality of infected cells. 

We were aware that this was an in vitro study that could only mimic a real condition of infection; moreover, a limitation of our study was the employment of a single cell line (i.e., SH-SY5Y), which was not in co-culture with other cell types that could better mimic the orofacial region, although the analysis of infection in different cells in the same well would have been a trivial task. A recent study describing the transcriptomic of SH-SY5Y confirmed that their expression profile showed a similar enrichment of biological processes with that of dorsal root ganglia neurons [29]; considering the latency of HSV-1 in neurons from trigeminal ganglia [1], SH-SY5Y could reasonably be considered as a good model in pain [29], and also in virology research.

Although further research is needed to understand the mechanism of action of the antiviral effect of blue PBMT, we would suggest a possible translation of the PBMT, at the protocols we set up, in the clinical setting for the treatment of herpes labialis. 

Since herpes labialis can affect the pediatric population [3], we deem that children can particularly benefit from the use of laser light, which is a painless, economic and effective procedure without side effects, to avoid or reduce the employment of antiviral pharmacological drugs.

## Data Availability

All the data used to support the findings in this study were included in the article.

## Supplementary Materials

The following are available online at https://www.mdpi.com/article/10.3390/life12010055/s1. Figure S1. (A) HSV-1 DNA quantity after 24 or 48 hours from inoculation with the different serial dilutions of HSV-1 employed. The viral DNA was expressed as Log10 viral copies/ml. (B) The viabil-ity of cells after 24 or 48 hours from inoculation with the different serial dilutions of HSV-1 em-ployed. The results are expressed as percentage respect to the not treated (NT) cells; Figure S2. Cells viability after 24 h from the irradiation with different PBMT protocols.
