# Supplementary material for: Blue Laser Light Counteracts HSV-1 in the SH-SY5Y Neuronal Cell Model of Infection
Source: Life (Basel). 2022 Jan 1;12(1):55. doi: 10.3390/life12010055 (PMC8778157; doi:10.3390/life12010055)
Supplement: Supplementary file 1 [file life-12-00055-s001.zip › life-1493091-SI.pdf]

life

MDPI

Supplementary Materials: Blue Laser Light Counteracts HSV-1 in SH-SY5Y Neuronal Cell Model of Infection

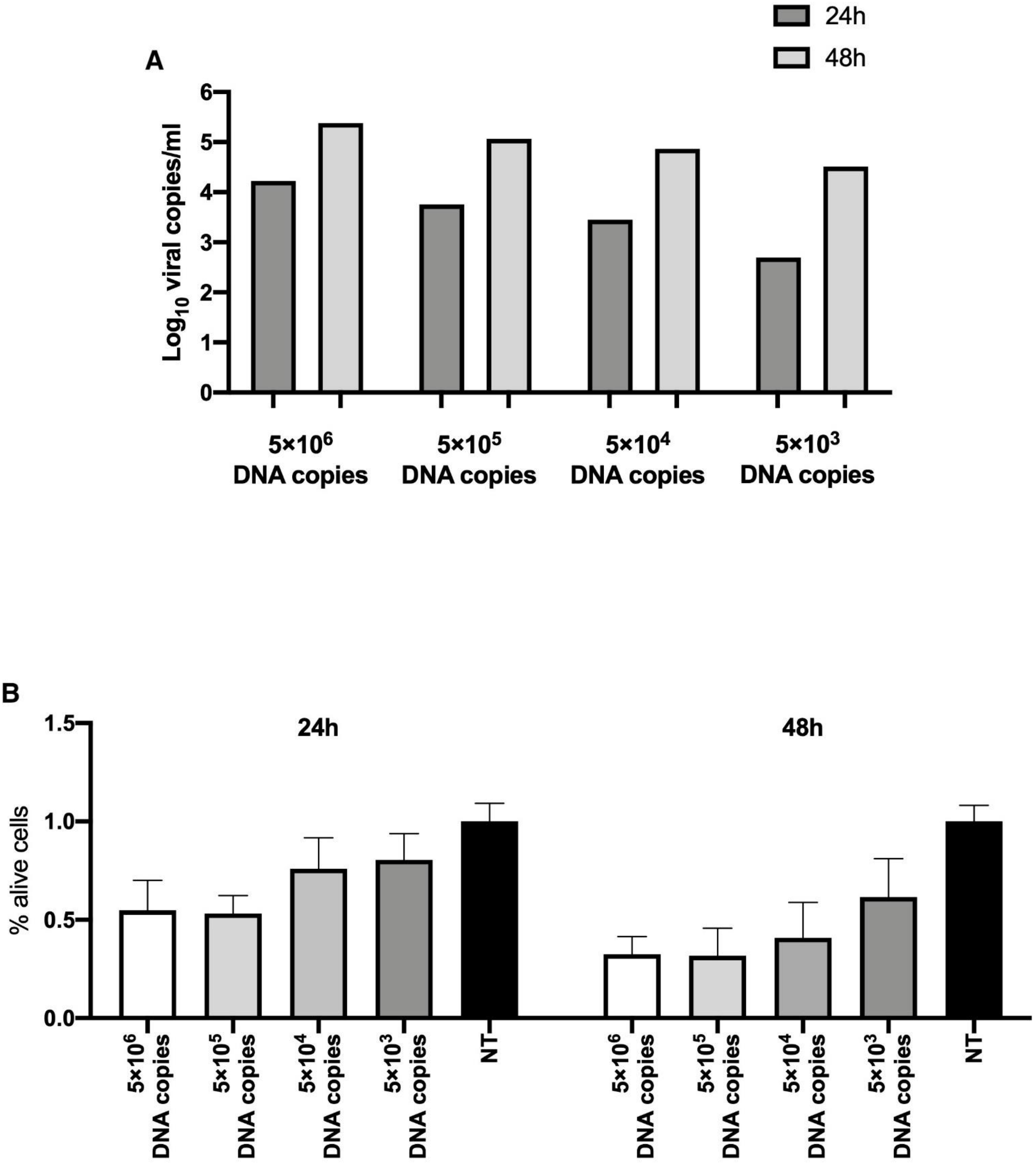

Figure S1. (A) HSV-1 DNA quantity after 24 or 48 hours from inoculation with the different serial dilutions of HSV-1 employed. The viral DNA was expressed as Log_{10} viral copies/ml. **(B)** The viability of cells after 24 or 48 hours from inoculation with the different serial dilutions of HSV-1 employed. The results are expressed as percentage respect to the not treated (NT) cells.

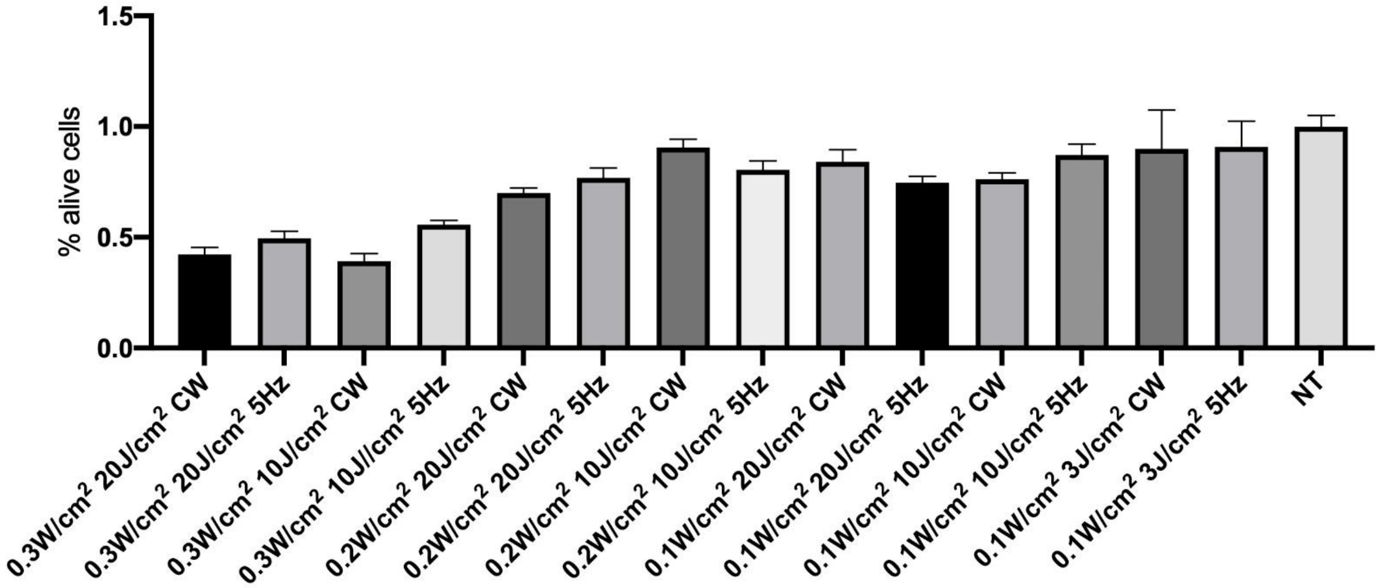

Figure S2. Cells viability after 24 hours from the irradiation with different PBMT protocols. The results are expressed as percentage respect to the not treated (NT) cells.
